# Editorial: Brain Hypoxia and Ischemia: New Insights Into Neurodegeneration and Neuroprotection

**DOI:** 10.3389/fnins.2019.00770

**Published:** 2019-07-25

**Authors:** Natalia N. Nalivaeva, Elena A. Rybnikova

**Affiliations:** ^1^Department of Physiology and Pathology of CNS, I.M. Sechenov Institute of Evolutionary Physiology and Biochemistry, Russian Academy of Sciences, Saint Petersburg, Russia; ^2^Department of Regulation of Brain Neuronal Functions, Faculty of Biological Sciences, School of Biomedical Sciences, University of Leeds, Leeds, United Kingdom; ^3^I.P. Pavlov Institute of Physiology, Russian Academy of Sciences, Saint Petersburg, Russia

**Keywords:** Alzheimer's disease, ischemic tolerance, hypoxic preconditioning, mitochondria, fatty acids, miRNAs, nanoparticles, prenatal hypoxia

Impaired oxygen supply (hypoxia) or reduced blood flow (ischemia) to the brain causes significant metabolic changes in neuronal and non-neural cells. It first leads to a rapid change in membrane lipid composition and enzyme activities and then to long-term changes in gene expression and levels of protein synthesis. They are often considered as major factors leading to cognitive impairment, seizures, and other neurological disabilities. The data accumulated to date suggest that vascular factors and reduced levels of oxygen supply to the brain are linked with the pathogenesis of various neurodegenerative disorders, in particular of Alzheimer's disease (AD), and can affect their progression.

Importantly, the central nervous system can withstand cerebral hypoxia or ischemia for a limited amount of time, a phenomenon called primary hypoxic–ischemic tolerance. With an appropriate time interval and dosage, when a non-injurious hypoxic exposure (known as preconditioning) is performed, tolerance can be increased and cells protected against lethal hypoxia exposures. Furthermore, the hypoxic preconditioning-induced neuronal tolerance appears to be universal and represents increased resistance not only to hypoxic/ischemic insults but also to other injurious factors including various types of stress.

The Research Topic “Brain hypoxia and ischemia: new insights into neurodegeneration and neuroprotection” evaluates recent progress in our understanding of the effects of hypoxia and ischemia on the brain at the molecular, morphological, and physiological levels. It also focuses on therapeutic avenues that are currently being developed to protect the brain and reduce pathology under various types of external and internal hypoxic challenge.

Taking into account the Developmental Origins of Health and Disease hypothesis (DOHaD) originally outlined for fetal programming of coronary heart diseases and later extended to mental health disorders, the review article by Nalivaeva et al. presents a detailed analysis of the effects of prenatal hypoxia during pregnancy on fetal brain development and increased risk of neurodegeneration in later life. It specifically outlines the importance of epigenetic reprogramming during embryonic development in response to various adverse environmental factors, which makes the organism vulnerable for development of neurological disorders, in particular AD. The authors evaluate various animal models for studying the consequences of prenatal hypoxia on brain physiology and biochemistry. A related research paper by Vasilev et al. gives an experimental example of how maternal hypoxia in certain periods of rat embryogenesis affects neuronal cell migration and formation of the brain structures involved in development of motor reactions and memory in the offspring in later life resulting in early cognitive deficit. In relation to AD pathogenesis, Kerridge et al. present cellular data on the effects of hypoxia on expression of the major amyloid-degrading enzyme neprilysin whose role in AD pathogenesis has recently been shown both in human and animal studies. These data explain how prenatal hypoxia can lead to reduced levels of the amyloid-precursor protein C-terminal fragment AICD which was shown to regulate neprilysin expression predisposing to AD. Furthermore, systemic hypoxia caused by carbon monoxide poisoning was also shown to promote Parkinson's disease as outlined in the article by Chang et al.

Discussing the response of organisms to reduced oxygen levels, Lukyanova and Kirova give a detailed analysis of the changes in energy metabolism in cells, especially in cortical neurons, which are the most vulnerable cells to reduced oxygen supply. They describe the role of mitochondria in development of immediate and delayed molecular mechanisms for adaptation to hypoxic challenge by reprogramming the respiratory chain function and switching from oxidation of NAD-related substrates (complex I) to succinate oxidation (complex II). They link succinate-related energy synthesis with stabilization of HIF-1α and initiation of its transcriptional activity which underlie long-term adaptation reactions.

In this regard the data presented in the research article by Brose et al. also reveal a novel neuronal specific pathway for adaptation to hypoxia through increased fatty acid biosynthesis. They have suggested that activation of fatty acid synthesis maintains reduction potential and reduces lactoacidosis in neuronal cells under hypoxia. Their work clearly demonstrates that fatty acids may serve as hydrogen acceptors under hypoxia supporting oxidation reactions including anaerobic glycolysis.

Biochemical changes which accompany brain ischemia often lead to accumulation of a toxic intermediate of methionine metabolism, homocysteine. Its detrimental effect on neuronal cells is underlined by accumulation of reactive oxygen species (ROS) and post-translational modifications of proteins via homocysteinylation and thiolation. In their review paper, Lehotský et al. summarize the effect of ischemia on intracellular signaling, especially in the mitogen-activated protein kinase (MAPK) protein pathways following ischemic injury. They provide evidence for the interplay and tight integration between ERK and p38 MAPK signaling mechanisms in response to homocysteine and also in association with ischemia and ischemic preconditioning challenge in the rat brain.

Summarizing the results of studying the molecular mechanisms and physiological responses of the organism to repeated mild hypoxia, Rybnikova and Samoilov discuss the effectiveness of specially designed hypoxic pre- and post-conditioning treatments. They demonstrate beneficial effects of these treatments not only on the outcome of the episodes of severe hypoxia but also on the reactions of individuals to various factors of a psycho-emotional nature. The mechanisms of hypoxic preconditioning which mobilize the defense processes and lead to development of brain tolerance are multileveled. They involve not only activation of intracellular cascades and changes in expression of multiple regulatory proteins in susceptible brain areas but also to modifications of the hypothalamic-pituitary-adrenal endocrine axis regulating various functions in the organism. A special role in these processes belongs to the epigenetic regulation of gene expression. In particular, changes in histone acetylation lead to chromatin remodeling which ensure access of pro-adaptive transcription factors activated by preconditioning to the promoters of target genes.

Another approach to increase brain tolerance as suggested by Horowitz et al. is activation of neuroprotective mechanisms following prolonged exposure to high ambient temperatures (heat acclimation). It alters molecular programs underlying cross-tolerance and enhances “on-demand” protective pathways evolved during acclimation. The protection achieved is long lasting and limits the need for de novo recruitment of cytoprotective pathways upon exposure to novel stressors. In particular using mouse and rat acclimated phenotypes, it was shown that the impact of heat acclimation is beneficial after traumatic brain injury as well as in global hypoxia models.

The emerging role of small non-coding RNA molecules (miRNAs) in regulation of brain and cellular functions also suggests their involvement in the response of organisms to hypoxia. Their neuroprotective potential under hypoxic conditions as discussed by Minhas et al. involves regulation of genes in oxygen and glucose deprived brain areas and is associated with the circadian rhythms. The authors suggest that alternate breathing or yogic intervention techniques can be considered as important non-invasive measures to protect the brain against hypoxia-associated pathology and discuss its efficacy for treatment of such neurodegenerative disease as AD.

Currently there are various pharmacological compounds which are neuroprotective against the damaging action of hypoxia and ischemia and they are widely discussed in various sections of the articles of this Research Topic. Special attention has been paid to the mechanisms of the protective action of an alkaloid sinomenine from a Chinese medicinal herb *Sinomeniumacutum* in a traumatic brain injury model. As demonstrated by Yang et al. this compound exerts its effect by activating the Nrf2–antioxidant response element pathway. Another neuroprotective approach against hypoxia-ischemic insult in newborns by cannabidiol treatment in association with hypothermia has been evaluated by Lafuente et al. They demonstrated the additive effects of both therapeutic factors resulting in reduced excitotoxicity, inflammation, oxidative stress, and overall cell damage in the brain if applied shortly after the insult.

Addressing an important problem of the delivery of neuroprotective or diagnostic compounds to the brain, Panagiotou and Saha discuss application of synthetic nanoparticles, which can cross the blood-brain barrier without compromising its integrity. They describe them as efficient carriers of therapeutic compounds designed for treatment of ischemic stroke.

This Research Topic clearly demonstrates the progress in our understanding of the molecular mechanisms underlying hypoxic-ischemic cell damage and the mechanisms of brain tolerance. However, it also makes clear that further studies of the complex interactions between multiple molecular pathways involved in epigenetic reprogramming of gene expression, energy metabolism, cell signaling, lipid- and protein homeostasis are needed to develop effective neuroprotective therapeutic measures against brain pathologies caused by insufficient oxygen supply to the brain.

## Author Contributions

NN and ER reviewed the submissions and wrote the editorial.

### Conflict of Interest Statement

The authors declare that the research was conducted in the absence of any commercial or financial relationships that could be construed as a potential conflict of interest.

